# Reproductive Isolation and Ecological Niche Partition among Larvae of the Morphologically Cryptic Sister Species *Chironomus riparius* and *C. piger*

**DOI:** 10.1371/journal.pone.0002157

**Published:** 2008-05-14

**Authors:** Markus Pfenninger, Carsten Nowak

**Affiliations:** Abteilung Ökologie & Evolution, Johan Wolfgang (J.W.) Goethe-Universität, Frankfurt, Germany; Stanford University, United States of America

## Abstract

**Background:**

One of the central issues in ecology is the question what allows sympatric occurrence of closely related species in the same general area? The non-biting midges *Chironomus riparius* and *C. piger*, interbreeding in the laboratory, have been shown to coexist frequently despite of their close relatedness, similar ecology and high morphological similarity.

**Methodology/Principal Findings:**

In order to investigate factors shaping niche partitioning of these cryptic sister species, we explored the actual degree of reproductive isolation in the field. Congruent results from nuclear microsatellite and mitochondrial haplotype analyses indicated complete absence of interspecific gene-flow. Autocorrelation analysis showed a non-random spatial distribution of the two species. Though not dispersal limited at the scale of the study area, the sister species occurred less often than expected at the same site, indicating past or present competition. Correlation and multiple regression analyses suggested the repartition of the available habitat along water chemistry gradients (nitrite, conductivity, CaCO_3_), ultimately governed by differences in summer precipitation regime.

**Conclusions:**

We show that these morphologically cryptic sister species partition their niches due to a certain degree of ecological distinctness and total reproductive isolation in the field. The coexistence of these species provides a suitable model system for the investigation of factors shaping the distribution of closely related, cryptic species.

## Introduction

Competition for resources will generally be most severe among closely related species, because they tend to have, due to their shared phylogenetic history, the most similar demands [1], [2]. It is widely assumed that the sympatric coexistence of sibling or sister species requires some sort of resource partitioning under resource-limited conditions [3]–[5], but see [6]. This “limiting similarity” concept [7] may not hold under certain, narrowly defined circumstances [8], [9], but these instances are believed to be rather the exception from the rule [8] . Hence, testing for differences in the realised ecological niche will consequently be the first logical step in order to explain the coexistence of similar, closely related species. However, closely related species often tend to be morphologically similar for the same reason they are ecologically alike [10]. Therefore, proper species delimitation and unequivocal recognition in field studies are a necessary prerequisite, often requiring molecular methods [11] .

The dipteran midges *Chironomus riparius* Meigen 1804 (synonym *C. thummi*, respectively *C. thummi thummi*) and *Chironomus piger* Strenzke 1959 (synonym *C. thummi piger*) are sister taxa [12], [13]. Larvae of both species are widely distributed in small streams, ditches, ponds and puddles throughout the holarctic [14]. The life cycle of *C. riparius* and *C. piger* consists of four larval stages, a short pupal stage and the adult midge. Adults form large mating swarms. A few days after hatching, female midges usually produce a single egg mass containing several hundreds of eggs. The larvae hatch after a few days, and the whole life cycle may be completed within four weeks. Depending on the water temperature, both species are usually multivoltin, with a first generation emerging early in spring and the final generation swarming around late autumn [15]. The overwintering generation consists solely of later larval stages (L3, L4). The species are often dominating the local *Chironomus* community [16]. In the study region they are frequently found together at the same sites [14], making the species pair an interesting model for the investigation of mechanisms enabling the sympatric coexistence of sibling species.

As the two sister taxa are morphologically cryptic, safe species discrimination was only possible only by analysis of polytene chromosomal structure in the past [17]. Despite their morphological similarity, genome size differs by 30%, mainly due to repetitive DNA [18]. Not only their taxonomic status regarding species or subspecies rank is unclear, also the reports on their degree of reproductive isolation are inconsistent. Some degree of prezygotic isolation in the field is warranted by differential swarming behaviour [19]. While some studies indicate that *C. riparius* and *C. piger* readily form viable and fully fertile interspecific hybrids in the laboratory [20], others estimate fertile hybrids in the wild to be effectively absent, due to fertility reductions caused by hybrid dysgenesis syndromes [21]. The actual degree of hybridisation and reproductive isolation in the field, however, has not yet been explored.

In this study we aimed to investigate distributional patterns of both species in an area where both species co-occur, and to reveal ecological factors that may have shaped the observed distribution. To this end, we investigated genetic differentiation between the species using mitochondrial and nuclear markers and related their relative abundance with environmental parameters. In particular, we answered successfully the following questions:

- What is the degree of reproductive isolation among *C. piger* and *C. riparius* in the field,
- is there a non-random spatial pattern of distribution and co-occurrence, and
- can we identify ecological parameters potentially structuring the species distribution?

## Results

### Species delimitation and identification

**Figure 1:**
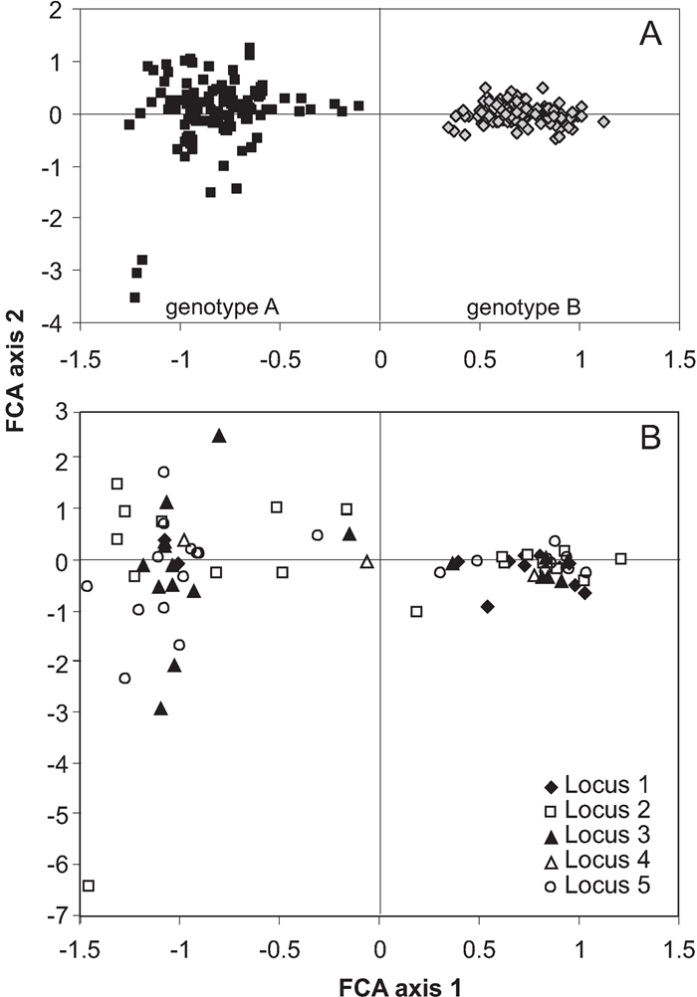
Factor score plot of A) individuals and B) microsatellite alleles on the first two axes of factorial correspondence analysis. A) The two obvious groups of *Chironomus* individuals separated along axis 1 are termed genotype A (grey squares) and genotype B (black diamonds). B) Contribution of microsatellite alleles, coded after locus. Most microsatellite alleles are typical if not exclusive for one of the two genotypes, only few alleles occur in similar proportions in both clusters.

**Figure 2:**
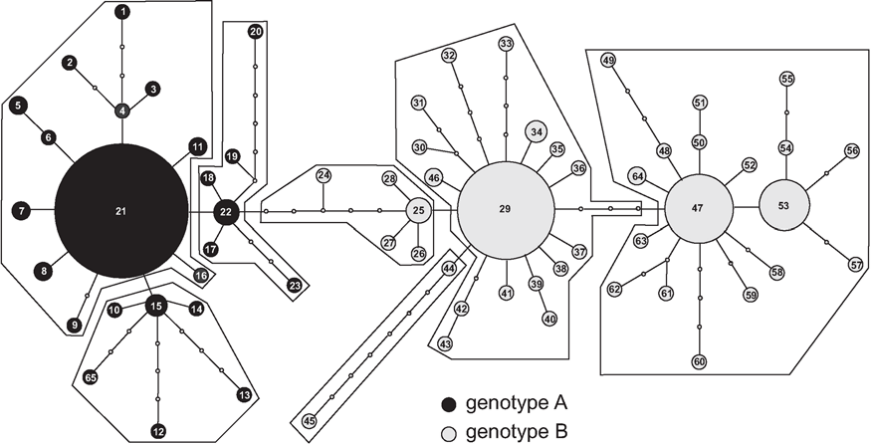
Statistical parsimony network of the mitochondrial COI haplotypes. The size of the circles is proportional to the number of individuals with the respective haplotypes. Polygons mark the 3-step clades. The nuclear genotype of the respective individuals is indicated (A = grey, B = black).

Two hundred sixty four individuals of *C. riparius/piger* were found at 34 sampling sites (Table 1). Microsatellite analysis detected a total of 76 alleles at the five loci (mean = 15.2, s.d. = 8.1). Factorial correspondence analysis on the microsatellite data revealed two distinct genotype clusters, termed A and B (Figure 1A). Their distinctness was due to both private alleles and frequency differences at all loci (Figure 1B). Identical results were obtained with other assignment methods like STRUCTURE (Pritchard *et al.*, 1999) (results not shown). The statistical parsimony network revealed two major haplotype groups, linked by six mutational steps. Plotting the two nuclear genotypes on the haplotypes of the respective individuals revealed a complete congruence with these two haplogroups (Figure 2). Polytene chromosome preparations identified genotype A (black symbols) consistently as *C. riparius* and genotype B (grey symbols) as *C. piger*.

**Table 1:** Sampling sites, their geographical position , number of *C. riparius/piger*individuals found on 1 m^2^ and number of individuals used for genetic analysis.

Site	Latitude	Longitude	Type of water body	Width (m)	Depth (m)	N *C.riparius/C. piger*	*N* µsats	*N* COI
ABR	49.4777	8.3207	ditch	1.3	0.3	1/1	2	1
BBB	49.3677	9.0267	creek	1.0	0.2	7/5	12	11
BBM	49.5268	8.6142	ditch	1.0	0.2	5/7	12	12
BGL	49.5121	8.2977	ditch	1.0	0.2	2/10	12	12
BMS	49.4380	8.3392	creek	1.0	0.2	-/8	5	8
EBO	49.2263	8.7006	ditch	1.5	0.3	1/-	-	2
FBD	49.4363	8.3249	creek	1.5	0.3	1/-	1	1
FBL	49.4246	8.3557	creek	1.0	0.1	8/-	8	8
GBH	49.3144	8.5307	ditch	1.0	0.1	7/3	10	10
GBR	49.2932	8.4151	creek	1.5	0.3	4/-	2	4
HBD	49.4499	8.6542	ditch	1.5	0.3	6/1	7	7
HBS	49.2730	8.3339	creek	0.6	0.2	8/-	7	8
KBH	49.3296	8.4913	ditch	2.0	0.3	25/-	25	24
KBK	49.2283	8.6405	creek	4.0	0.8	1/2	1	2
KBT	49.3313	8.5248	creek	4.0	0.6	1/2	2	1
KLB	49.2264	8.6429	creek	2.5	0.2	-/1	1	1
LBD	49.4145	8.3165	ditch	1.0	0.1	2/10	9	11
LGH	49.5431	8.3168	ditch	2.5	0.3	5/17	22	11
LGN	49.3291	8.6814	creek	4.0	0.5	1/-	-	1
MBD	49.4408	8.6674	creek	1.5	0.3	8/1	7	7
MBF	49.4710	8.2921	ditch	1.5	0.3	2/8	5	5
NBL	49.4996	8.2911	ditch	1.5	0.3	-/7	9	6
NBM	49.4919	8.2894	ditch	1.5	0.2	1/5	5	4
PBF	49.4363	8.3249	puddle	0.4	<0.1	-/9	9	7
PBR	49.3789	8.3512	puddle	0.4	<0.1	-/10	10	6
PFO	49.2137	8.6936	puddle	0.5	<0.1	1/6	7	5
RBD	49.4519	8.6422	creek	1.5	0.2	9/4	13	9
SBB	49.5579	8.3099	creek	3.0	0.4	2/4	6	5
SBR	49.2749	8.4628	ditch	2.5	0.3	-/1	1	-
SBS	49.5813	8.6471	creek	0.5	0.1	21/-	21	7
TAS	49.3025	8.7166	pond	10	1.5	-/2	-	2
TBN	49.3207	8.7079	pond	6.0	1.3	1/-	-	1
WBH	49.3145	8.3361	creek	4.0	0.4	2/1	3	1
WGH	49.3126	8.3947	creek	3.0	0.3	8/-	8	3
						Σ 139/125		

### Co-occurrence and population structure

**Figure 3:**
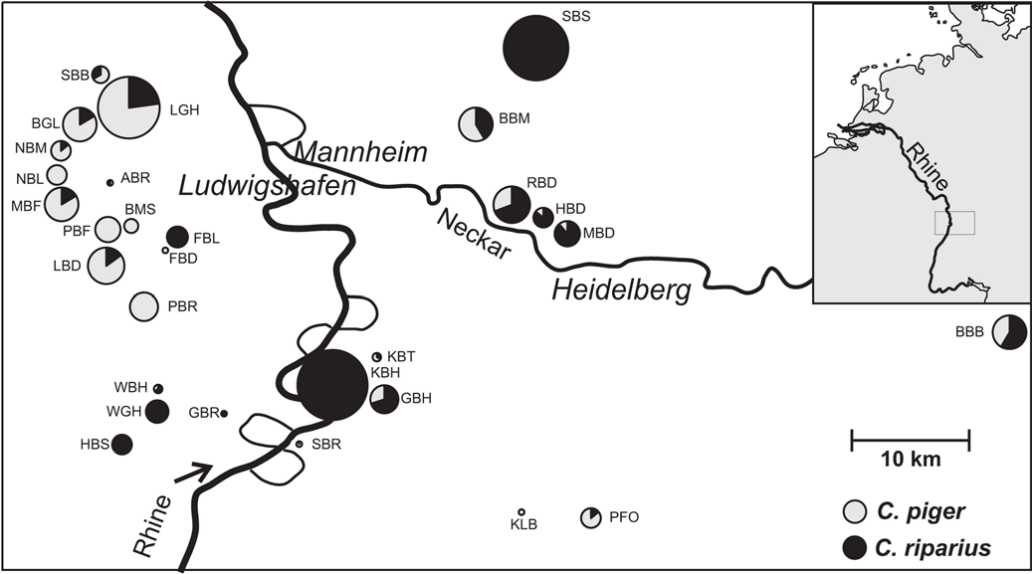
Map of the sampling points in the Rhine valley with pie charts of the relative frequency of *Chironomus riparius* (black) and *C. piger* (grey). The size of the circles corresponds to the number of individuals sampled.

**Figure 4:**
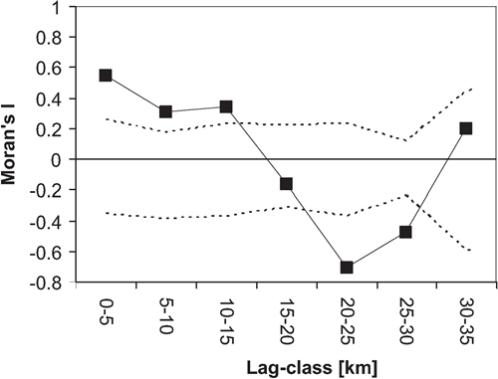
Spatial autocorrelation analysis of the relative *C. riparius* frequency for lag-classes of 5 km. Dashed lines indicate the 95% confidence interval of observed Moran's I being significantly different from zero, derived from 999 permutations.

At about half of the sampling sites containing *C. riparius* or *C. piger*, both species co-occurred in varying proportions (Figure 3). However, individuals of the species occurred less often together than expected by chance (Fisher's exact test χ^2^ = 160, d.f. = 22, p<0.0001). The relative frequency of a species at a given site was not independent from their frequency at surrounding sites. We found a significant spatial autocorrelation of sampling sites up to 15 km apart (Figure 4). A significant, albeit very weak genetic population structure within both species was detected. For *C. piger*, a Φ_ST_ of 0.027 was calculated, while the estimate for *C. riparius* was 0.046 (Table 2), indicating a high amount of gene-flow among sampling sites among individuals of each species.

**Table 2:** Population structure estimated with AMOVA of microsatellite data for *Chironomus piger* and *C. riparius*.

Source of variation	*d.f.*	SS	MS	Percent of total variance	Φ_ST_	*p*
Among *C. piger* sampling sites	10	38.673	3.867	3	0.027	0.040
Within sampling sites	85	265.460	3.123	97		
Total	95	304.133	6.990			
Among *C. riparius* sampling sites	8	44.717	5.590	5	0.046	0.010
Within sampling sites	91	334.972	3.681	95		
Total	99	379.689	9.271			

Significance of variance components was assessed with 9999 permutations.

### Environmental correlates of relative species frequencies

Of the 38 variables tested, six were significantly correlated (p<0.05, q<0.10) with the relative *C. riparius* frequencies: conductivity (r = −0.624), Nitrite (−0.611), CaCO_3_ (−0.725), precipitation in the wettest month (+0.672), and precipitation in the warmest quarter (+0.582) (Table 3). Multiple regression retained only precipitation in the wettest month (July) and in the warmest quarter as significant (May–July, Table 4).

**Table 3:** Pearson correlation coefficients (*r*) of environmental variables with relative frequencies of *C. riparius* and the respective *p* and *q* values for multiple comparisons.

variable	*r*	*p*	*q*
pH	0.155	0.566	0.354
Conductivity	*−0.624	0.010	0.048
Velocity	0.372	0.156	0.195
Temperature	−0.246	0.359	0.346
O_2_	0.418	0.108	0.178
Nitrate	0.169	0.531	0.354
Nitrite	*−0.611	0.012	0.048
Phosphate	0.119	0.662	0.378
Ammonium	−0.135	0.619	0.375
CaCO_3_	*−0.725	0.001	0.020
Chloride	−0.274	0.304	0.320
Organic content	0.087	0.750	0.388
>4mm	0.071	0.795	0.388
<4mm>2mm	0.244	0.363	0.346
<2mm>1mm	0.292	0.273	0.304
<1mm>630µm	0.170	0.530	0.354
<630µm>500µm	−0.127	0.638	0.375
<500µm>250µm	−0.226	0.400	0.354
<250µm	0.007	0.978	0.436
Annual Mean Temperature	0.201	0.455	0.354
Mean Monthly Temperature Range	0.006	0.982	0.436
Isothermality	−0.409	0.116	0.178
Temperature Seasonality	0.467	0.068	0.160
Max Temp. of Warmest Month	0.162	0.549	0.354
Min Temperature of Coldest Month	−0.082	0.763	0.388
Temp Annual Range	0.291	0.274	0.304
Mean Temp Wettest Quarter	0.209	0.438	0.354
Mean Temp Dryest Quarter	0.054	0.844	0.402
Mean Temp Warmest Quarter	0.209	0.438	0.354
Mean Temp Coldest Quarter	0.075	0.784	0.388
Annual Preciptiation	0.446	0.083	0.166
Prec Wettest Month	*0.672	0.004	0.040
Prec Driest Month	0.383	0.143	0.195
Prec Seasonality	0.164	0.544	0.354
Mean Prec Wettest Quarter	*0.582	0.018	0.051
Mean Prec Dryest Quarter	0.374	0.153	0.195
Mean Prec Warmest Quarter	*0.582	0.018	0.051
Mean Prec Coldest Quarter	0.193	0.474	0.354

Correlations with p<0.05 and q<0.1 are marked with asterisks.

**Table 4:** Multiple regression of environmental parameters significantly correlated with relative *C. riparius* frequencies (forward selection).

variable	β	b	*p*
Prec Wettest Month (July)	2.60	34.90	0.007
Mean Prec Wettest Quarter (May–July)	2.18	36.33	0.020
CaCO_3_	−0.69	−0.60	0.102
Conductivity	0.47	0.89	0.225
Nitrite	−0.20	−1.78	0.309

The regression was significant (N = 18, F_(5,12)_ = 6.7, *p* = 0.003, corr. r^2^ = 0.63).

## Discussion

### Chironomus riparius and C. piger behave as good species in the field

Microsatellite analysis showed the presence of two distinct genotype groups without intermediates, indicating complete reproductive isolation and the absence of putative hybrids (Figure 1A). Most alleles were specific to one of the cluster with only few alleles shared in similar proportions by the two taxa (Figure 1B). As only the lengths of PCR-fragments were scored, this partial overlap may be due to homoplasy or common ancestry. The results were so clear cut that the application of more sophisticated methods of hybrid detection (e.g. NewHybrids) was deemed unnecessary. The inference of reproductively isolated gene-pools is strengthened by the distinctness of the mitochondrial variation of the genotype groups (Figure 2), indicating long lasting isolation with absence of both current and past hybridisation [22]. Even though the generation of hybrids in the laboratory is possible to varying degrees [17], [21], both pre- and postzygotic isolation mechanisms [19], [21] seemed to have maintained complete reproductive isolation in the wild, despite the opportunity to interbred. Therefore, the two taxa conform to several species concepts, including the biological [10] at least in the area investigated and they should be consequently regarded as good species.

### Spatial repartition of C. riparius and C. piger along ecological gradients

The unequivocal species assignment by molecular markers showed that larvae of *C. riparius* and *C. piger* occurred not only in the same general area, but in about half of the cases at the same site (Figure 3). Still, they were found less often syntopically than expected from their overall abundance. The virtual absence of population structure on the spatial scale of the study (Table 2) suggests that this is not due to dispersal restrictions or geographical obstacles. It indicates rather competitive interaction, either present or past [23]. *C. piger* was dominant mainly in the west of the area, while *C. riparius* occurred more frequently in the east (Figure 3), as mirrored in the significant spatial autocorrelation of relative species frequency (Figure 4). This pattern corresponds to the correlation of the species' relative frequencies with parameters measuring the average amount of precipitation during the summer months (Table 3). The latter are climatic parameters which generally tend to be spatially autocorrelated. Other parameters that covaried significantly with relative species abundances were water chemistry variables (conductivity, nitrite and CaCO_3_, Table 3). Multiple regression retained only the precipitation variables (Table 4). This suggests that differential desiccation resistance, as observed in other *Chironomus* species [24], could have caused the observed patterns. However, the absolute differences in precipitation (see Appendix) are probably too small for a differential desiccation risk of the water bodies in the area. Therefore, the climate constitutes probably merely the ultimate cause for the differential species distribution. The amount of rain during the warm summer months with their increased evaporation determines the concentrations of nitrite and other ions in the shallow puddles and ditches both species inhabit. It has been shown that both high salinity and high nitrite concentrations impart larval development in *C. riparius/piger* [25], [26]. Our results indicate that *C. piger* occurs in areas with less summer rain and tolerates higher nitrite concentrations and conductivity than *C. riparius* (Table 3). Therefore, the proximate cause for the observed correlation of the species frequencies with summer precipitation is more likely the gradient of water chemistry variables during the time of highest larval abundance [27].

Even though the inferred spatial repartition along ecological gradients is rather a hypothesis in need to be confirmed by subsequent experiments in the laboratory, it has become evident that *C. riparius* and *C. piger* are ecologically not completely equivalent. Although this has already been suspected before [14], our study is the first to demonstrate ecological partitioning among the species pair quantitatively in the field. Studies on the ecological differentiation of other *Chironomus* species have revealed a range of mechanisms that structure coexistence in sympatry. Dietary niche separation among two profundal species from the *Chironomus plumosus*-group has been suggested by stable isotope analysis [28]. The same species were found to differ in emergence time, suggesting also temporal niche separation [29]. Perhaps the most impressive example of interspecies competition avoidance is the spatial repartition of temporary rain water puddles by *C. pulcher* and *C. imicola* into shaded and sunny regions on a very small scale [30].

Despite the demonstrated spatial repartition along ecological gradients of *C. riparius* and *C. piger*, we found a substantial number of sites where both species co-occurred, indicating a substantial overlap in the realised ecological niche. Possible, not mutually exclusive explanations for this pattern include: i) a substantial stochasticity in the dispersal/colonisation of the sites. Even though the oviposition choice in another *Chironomus* species is influenced by nitrogenous compounds and conspecific larvae [31], a high degree of randomness regarding environmental conditions is generally assumed in the community assembly of chironomids [27]. Also which species arrived first at a yet unoccupied site may crucially influence the outcome of subsequent competition [32]. ii) Temporally fluctuating environmental conditions may also prevent complete competitive exclusion [33]. iii) Interaction with other species. Several other species of *Chironomus* are present at most of the investigated sites [16], as well as other mud dwelling taxa with similar requirements. iv) the abundance in the neighbourhood possibly also influences the local abundance of *C. riparius* and/or *C. piger* [33].

As this study documents, *C. riparius* and *C. piger* provide a promising model for the investigation of factors shaping the distribution of closely related, cryptic species. Currently ongoing experimental and ecological genomic studies on this emerging model system will help to gain a deeper understanding of the processes and factors that shape the realised niche of closely related species in sympatry. Understanding the internal factors and constraints shaping their distribution and coexistence will contribute to our mechanistical understanding of the processes shaping biodiversity in ecological communities.

## Materials and Methods

### Sampling

The sampling area lies in the middle of the upper Rhine valley in a rectangle of roughly 40 by 60 km between 49°09′–49°33′N and 8°10′–8°13′E. It comprises the Rhine valley plain, in the west limited by the mountains of the Pfälzer Wald and in the east by the rising hills of the Odenwald range. The area is hydrologically characterised by the presence of many drainage ditches, slowly flowing small streams, temporary puddles, the oxbows and the main stream of the river Rhine.

The sampling took place from mid September to November 2004, thus sampling the over-wintering generation of *Chironomus* larvae [34]. The sampling period was scheduled in autumn in order to avoid the large fluctuations in abundance among species throughout summer. Moreover, sampling the hibernating larvae assemblage that will foster next years first generation presents the result of competition processes during the growth season [35].

Sampling took place as described in [12]. Briefly, potential *Chironomus* habitats were considered opportunistically within the study region, but we mainly focused on typical *Chironomus riparius*/*piger* habitats (small streams, creeks, and ditches with fine, muddy sediment). An area of 1×1 m was sampled with a 30×40 cm net of 0.5 mm mesh size. Due to the small size and low depth of most water bodies, we did not consider different areas within a water body during sampling. All *Chironomus* larvae instar stage found (instar stage 3 and 4), as identified by the presence of ventral tubuli, were brought alive into the laboratory. For the present study, we chose all thirty-four sampling sites where *C. riparius* and/or *C. piger* which had been identified earlier using a COI barcoding approach [16].

### DNA isolation and microsatellite analyses

Larvae were kept in the laboratory for at least 5 days without feeding, in order to remove potential PCR inhibiting substances from the gut [36]. Head and first body segments were removed for polytene chromosome analysis as described in [17]. Briefly, salivary glands were prepared from fresh larval tissue and fixed in 50% acetic acid. Chromosomes were stained in 2% orcein acetic acid for 15 min and fixed on glass slides for microscopical analysis. Remaining caudal tissue was homogenized in 700 µl standard CTAB buffer containing 0.1 mg/ml proteinase K. After digestion for at least 1 h at 62° C, chloroform/isoamyl alcohol 24:1 treatment was performed followed by 1 h precipitation at −20° C. DNA pellets were washed twice with ethanol 70% and resolved in 30 µl water.

Allelic variation was measured at five variable, unlinked microsatellite loci [37] for 255 individuals from 29 locations (Table 1). Microsatellite fragments were amplified as described in [37]. Amplified DNA fragments were diluted 1:25 prior to fragment length analysis (ALF sequencer, Pharmacia Biotech, Uppsala, Sweden) and alleles were scored using the ALFWIN 1.0 software (Pharmacia Biotech, Uppsala, Sweden).

### Genetic structure, mitochondrial haplotype phylogeny and species identification

Factorial correspondence analysis (FCA) was applied on multilocus genotypes to explore the distribution of genetic variation graphically (GENETIX 4.04 software, [38]. Genetic population structure was assessed using the AMOVA approach [39] as implemented in the Excel add-in GenAlEx [40]. For this analysis only sampling sites with at least seven conspecific individuals were taken into account. *C. riparius/piger* COI haplotypes were identified from [16] (GenBank Accession numbers DQ910547-DQ910729). The phylogeny of the COI haplotypes was inferred using statistical parsimony (SP) [41]. The SP network was constructed with the program TCS v. 1.21 [42]. Nesting of clades followed the rules given in [43] and [44]. Inferred reproductively isolated entities were taxonomically identified using polytene chromosome preparations of a subset of individuals [17].

### Co-occurrence

We used a Fisher's exact test (10^6^ permutations) to investigate whether the co-occurrence of the identified taxa was random. Spatial patterns of the relative frequency of *C. riparius* and relevant environmental parameters at the sample sites with at least seven individuals found were inferred with spatial autocorrelation analysis. Seven mutually exclusive lag classes of 5000 m width were used to compute Moran's *I* spatial correlation coefficient for each class. Statistical significance of Moran's *I* was assessed with 999 Monte Carlo permutations. The Excel Add-in RookCase version 0.99 [45] was used for the calculations.

### Physico-chemical and climatic characterisation of sampling sites

Thirty-eight ecological parameters were recorded in order to characterize abiotic habitat conditions at the respective sampling size. These parameters were chosen to cover a wide range of ecological conditions known to influence freshwater communities, and the distribution of chironomid species in particular. Recorded characteristics include physicochemical parameters [46], [47], sediment composition [48], climatic conditions [49], and structural habitat characteristics (e.g., size and depth of water body).

For the determination of sediment organic content, measured as loss on ignition, approximately 30 g of sediment sample were dried at 60°C for three days and weighed subsequently. Samples were then muffled at 550°C for 4 h, followed by determination of percentage weight loss.. For the identification of relative particle size composition of the samples, 150 g of homogenised sediment were washed through six sieves with decreasing mesh size and the content of each sieve was dried and weighted.

Conductivity, pH, water temperature and O_2_ saturation were measured with a WTW Multi 340i multimeter at each sampling site. Ammonium, nitrite and phosphate concentrations were calorimetrically determined using Aquamerk® quicktests. Chloride, CaCO_3_ and nitrate concentrations were measured with colour tests (Merkoquant®). The stream velocity was measured using an AMR ALMEMO® device.

Nineteen biologically meaningful climatic parameters were extracted for each sampling site from the BIOCLIM environmental layers with a spatial resolution of 0.5 min, implemented in the computer program DIVA-GIS version 4.2 [50]. Mean, median, standard deviation, minimum and maximum values for the recorded parameters are given in Appendix S1.

### Statistical analysis

Means, standard deviations, median, minimum and maximum values for all 38 variables taken into account are given in the Appendix. All data with the exception of pH were either log_10_ (x+1; continuous variables) or arcsin (percentages) transformed to conform to the underlying assumptions of normality and heteroscedasticity in subsequent analyses. We calculated Pearson's correlation coefficients (*r*) between the relative *C. riparius* frequencies and all respective variables. Due to the multitude of comparisons, we calculated a q value for each test to estimate the minimum false discovery rate which is incurred when calling that test significant. Variables with p values<0.05 and q values<0.10 in correlation analysis were retained for a multiple regression (forward selection).

## Supporting Information

Appendix S1 Recorded environmental parameters.
(0.11 MB DOC)
