# Supplementary material for: Reproductive Isolation and Ecological Niche Partition among Larvae of the Morphologically Cryptic Sister Species Chironomus riparius and C. piger
Source: PLoS One. 2008 May 14;3(5):e2157. doi: 10.1371/journal.pone.0002157 (PMC2364647; doi:10.1371/journal.pone.0002157)
Supplement: Appendix S1 — Recorded environmental parameters. (0.11 MB DOC) [file pone.0002157.s001.doc]

Appendix : Recorded environmental parameters.

| Variabl  e set | variable | abbreviation | unit | mean | s.d. | median | min | max |
| --- | --- | --- | --- | --- | --- | --- | --- | --- |
| Bioclimate n = 19 | Altitude over sea level | ALTITUDE | m | 110*.*00 | 30*.*06 | 100*.*50 | 93*.*00 | 242*.*00 |
|  | Annual mean temperature | ANNTEMP | °C | 10*.*22 | 0*.*14 | 10*.*22 | 9*.*84 | 10*.*43 |
|  | Mean monthly temperature range | TEMPRANGE | °C | 8*.*67 | 0*.*04 | 8*.*67 | 8*.*58 | 8*.*78 |
|  | Isothermality | ISOTERM | °C | 32*.*95 | 0*.*18 | 32*.*94 | 32*.*67 | 33*.*27 |
|  | Mean annual temperature seasonality | SEASONTEMP | °C | 676*.*30 | 3*.*86 | 676*.*73 | 668*.*98 | 682*.*38 |
|  | Maximum temperature of warmest month | MAXWARM | °C | 24*.*69 | 0*.*20 | 24*.*65 | 24*.*10 | 25*.*00 |
|  | Mean annual minimum temperature of coldest month | MINCOLD | °C | -1*.*63 | 0*.*15 | -1*.*60 | -2*.*30 | -1*.*50 |
|  | Mean annual temperature annual range | ANNRANGE | °C | 26*.*31 | 0*.*11 | 26*.*35 | 26*.*10 | 26*.*50 |
|  | Mean annual temperature of wettest quarter | MTEMPWET | °C | 18*.*46 | 0*.*17 | 18*.*48 | 18*.*05 | 18*.*68 |
|  | Mean annual temperature of driest quarter | MTEMPDRY | °C | 3*.*07 | 0*.*14 | 3*.*10 | 2*.*53 | 3*.*27 |
|  | Mean annual temperature of warmest quarter | MTEMPWARM | °C | 18*.*46 | 0*.*17 | 18*.*48 | 18*.*05 | 18*.*68 |
|  | Mean annual temperature of coldest quarter | MTEMPCOLD | °C | 1*.*84 | 0*.*13 | 1*.*86 | 1*.*37 | 1*.*98 |
|  | Mean annual precipitation | ANNPREC | mm | 659*.*45 | 23*.*09 | 664*.*50 | 617*.*00 | 701*.*00 |
|  | Mean annual precipitation in the wettest month | PRECWET | mm | 75*.*97 | 4*.*06 | 77*.*00 | 69*.*00 | 83*.*00 |
|  | Mean annual precipitation in the driest month | PRECDRY | mm | 41*.*89 | 1*.*71 | 42*.*00 | 39*.*00 | 45*.*00 |
|  | annual Precipitation seasonality | SEASONPREC | without dimension | 20*.*11 | 0*.*89 | 19*.*73 | 18*.*95 | 21*.*77 |
|  | Mean annual precipitation in the wettest quarter | MPRECWET | mm | 211*.*76 | 8*.*74 | 214*.*00 | 197*.*00 | 228*.*00 |
|  | Mean annual precipitation in the driest quarter | MPRECDRY | mm | 130*.*84 | 4*.*55 | 132*.*00 | 122*.*00 | 139*.*00 |
|  | Mean annual precipitation in the warmest quarter | MPRECWARM | mm | 211*.*76 | 8*.*74 | 214*.*00 | 197*.*00 | 228*.*00 |
|  | Mean annual precipitation in the coldest quarter | MPRECCOLD | mm | 141*.*39 | 4*.*14 | 142*.*50 | 134*.*00 | 149*.*00 |
| Physico-chemical parameters n = 19 | pH |  |  | 7*.*70 | 0*.*39 | 7*.*68 | 6*.*87 | 8*.*70 |
|  | Organic content of sediment |  | % loss on ignition | 16*.*03 | 12*.*91 | 12*.*57 | 3*.*06 | 85*.*80 |
|  | Particles >4mm | 4MM | % weight | 12*.*76 | 12*.*40 | 10*.*00 | 0*.*67 | 66*.*67 |
|  | Particles <4mm>2mm | 2MM | % weight | 8*.*86 | 5*.*80 | 7*.*33 | 0*.*67 | 28*.*00 |
|  | Particles <2mm>1mm | 1MM | % weight | 9*.*38 | 5*.*20 | 10*.*00 | 0*.*67 | 22*.*00 |
|  | Particles <1mm>630µm | 630UM | % weight | 11*.*35 | 6*.*97 | 9*.*33 | 3*.*33 | 34*.*00 |
|  | Particles <630µm>500µm | 500UM | % weight | 18*.*23 | 12*.*19 | 16*.*00 | 0*.*00 | 50*.*67 |
|  | Particles <500µm>250µm | 250UM | % weight | 20*.*60 | 12*.*21 | 19*.*33 | 0*.*67 | 50*.*67 |
|  | Particles <250µm | <250UM | % weight | 18*.*86 | 18*.*95 | 10*.*00 | 1*.*33 | 80*.*00 |
|  | Conductivity |  | µS/cm | 845*.*76 | 455*.*45 | 886*.*50 | 118*.*00 | 2030*.*00 |
|  | Stream velocity |  | m/s | 0*.*06 | 0*.*10 | 0*.*02 | 0*.*00 | 0*.*41 |
|  | Water temperature |  | °C | 9*.*25 | 2*.*88 | 8*.*55 | 4*.*40 | 15*.*50 |
|  | O_2_ |  | mg/l | 6*.*93 | 2*.*62 | 7*.*05 | 0*.*60 | 11*.*80 |
|  | Nitrate |  | mg/l | 21*.*97 | 81*.*55 | 0*.*00 | 0*.*00 | 500*.*00 |
|  | Nitrite |  | mg/l | 0*.*16 | 0*.*18 | 0*.*10 | 0*.*00 | 1*.*00 |
|  | Phosphate |  | mg/l | 0*.*79 | 0*.*72 | 0*.*50 | 0*.*00 | 3*.*00 |
|  | Ammonium |  | mg/l | 1*.*07 | 1*.*73 | 0*.*50 | 0*.*00 | 7*.*00 |
|  | CaCO_3_ |  | mg/l | 269*.*81 | 134*.*39 | 373*.*80 | 53*.*40 | 462*.*80 |
|  | Chloride |  | mg/l | 16*.*03 | 12*.*91 | 12*.*57 | 3*.*06 | 85*.*80 |
